# Biocidal Polymers: Synthesis, Characterization and Antimicrobial Activity of Bis-Quaternary Onium Salts of Poly(aspartate-*co*-succinimide)

**DOI:** 10.3390/polym13010023

**Published:** 2020-12-23

**Authors:** Mohamed H. El-Newehy, Meera Moydeen A., Ali K. Aldalbahi, Badr M. Thamer, Yehia A.-G. Mahmoud, Hany El-Hamshary

**Affiliations:** 1 Department of Chemistry, College of Science, King Saud University, Riyadh 11451, Saudi Arabia; meeranis@yahoo.co.in (M.M.A.); aaldalbahi@ksu.edu.sa (A.K.A.); bthamer@ksu.edu.sa (B.M.T.); helhamshary@ksu.edu.sa (H.E.-H.); 2 Department of Chemistry, Faculty of Science, Tanta University, Tanta 31527, Egypt; 3 Department of Botany and Microbiology, Faculty of Science, Tanta University, Tanta 31527, Egypt; yehia.mahmoud@science.tanta.edu.eg

**Keywords:** quaternary salts, antimicrobial activity, water-soluble polymers, poly(aspartate-*co*-succinimide)

## Abstract

Microbial multidrug resistance presents a real problem to human health. Therefore, water-soluble polymers based on poly(aspartate-*co*-succinimide) were synthesized via reaction of poly(aspartate-*co*-succinimide) with bis-quaternary ammonium or quaternary salts. The resultant copolymers were characterized by various techniques such as FTIR, TGA, ^1^HNMR, ^13^CNMR and elemental microanalysis. Antimicrobial activities of the new onium salts were investigated against *Escherichia coli, Pseudomonas aeruginosa, Staphylococcus aureus*, *and Salmonella typhi,* and the fungi; *Candida albicans,* *Aspergillus niger*, *Cryptococcus neoformans* and *Aspergillus flavus* by agar diffusion method. Antimicrobial activity was studied in terms of inhibition zone diameters, in addition to the estimation of minimal inhibitory concentration (MIC) of the prepared compounds. *A. niger* and *E. coli* were the most affected microorganisms among the tested microorganisms with an inhibition zone of 19–21 (mm) in case of biocides, (V) and (VII). The obtained results showed that the quaternary onium salts have higher activity compared to the aspartate copolymer with MIC concentrations of 25 mg/mL for (VII) and (V) and 50 mg/mL for (VI) and (IV).

## 1. Introduction

In recent years, the management of infectious disease has become a challenge, and microbial infections are a top concern [1]. Biomedical fields such as hospitals, healthcare products, medical and surgical utensils, food preservation, and household sanitization are facing the problem of contamination by microbes in the environment [2]. *Escherichia coli* as one of the fecal coliform family, is the most common causes of fecal contamination than other fecal coliforms. Common microbes in the environment—bacteria, yeast, and fungi—are making a severe impact on human health [3,4,5]. To control such contaminations, a variety of antimicrobial agents such as antibiotics [6], nanoparticles [7] and biofilms [8] have been used.

However, the development of antimicrobial resistance to certain antibiotics opens the way to search for new antimicrobial agents to avoid problems associated with the use of antibiotics in controlling the growth of such microbes [9]. Resistance to antibiotics is mostly observed in Gram-negative bacteria by interacting with the drugs causing amplification of mortality and increasing the cost of treatment [10]. In addition, many suspect that the used number of drugs may cause residual toxicity and have hazardous effects on the environment. Additionally, the nanoparticles are small, and can be inhaled easily by breathing from the environment. These materials can first affect the respiratory tract via deposition in the human lungs and then move to other organs of the body like the liver, brain, etc. [11].

To overcome this problem, the application of antimicrobial biocidal polymers may replace antibiotic drugs [12,13]. There is a wide range of different polymeric materials available naturally and can be prepared synthetically. However, some of these have significant low antimicrobial action against the drug-resistant microorganisms. Due to their reduced residual toxicity, increased lifetime and enhanced antimicrobial properties, such polymers can be used in different fields like food packaging and medicine [14,15].

Among the reported polymeric materials, water-soluble polymer, poly(aspartate), is a biocompatible and biodegradable polymer, which can be used in pharmaceutical and food industries due to its nontoxicity and mutagenic effects [16,17]. The derivatives of poly(aspartic acid) like poly(succinimide) and poly(hydroxyethyl aspartamide) have also acquired more attention due to their biodegradability in nature [18].

Noteworthy, quaternary onium salts are positively charged materials commonly used as disinfectants in many food industries. The positive charge of quaternary onium salts has great potential to react with a negative charge of most microbes in the environment. Moreover, polymer-based onium salts are generally used as antimicrobial agents, which can reduce the residual toxicity and enhance the antimicrobial efficacy [14,19]. Liu et al. prepared the polyionene with *N,N,N′,N′*-tetramethyldiamine (TMEDA), which has shown excellent antimicrobial properties with non-hemolytic properties [20].

In the current study, we developed new antimicrobial water-soluble polymers based poly(aspartate-*co*-succinimide) through reaction with different quaternary ammonium and phosphonium salts. The antimicrobial action of the new onium salts was investigated against different types of pathogenic microbes (*Escherichia coli, Pseudomonas aeruginosa, and Staphylococcus aureus*, *Salmonella typhi,* and the fungi; *Aspergillus niger*, *Candida albicans, Cryptococcus neoformans* and *Aspergillus flavus*) by estimation of inhibition zone diameters as well as the minimum inhibitory concentration (MIC).

## 2. Experimental

### 2.1. Materials

Copoly(aspartate succinimide) 1:1 residual ratio was purchased from Folia, Inc. (Birmingham, AL, USA). *N,N,N′,N′*-Tetramethylethylenediamine (TMEDA), tetraethylammonium bromide (TEAB) and methyltriphenylphosphonium bromide (MTPPB) were supplied by Sigma-Aldrich, Steinheim, Germany. All solvents were used as supplied without additional purification.

### 2.2. Synthesis of N,N,N′,N′-Tetramethylethylenediamine Dihydrochloride (TMEDA.2HCl) (II)

**Scheme 1:**
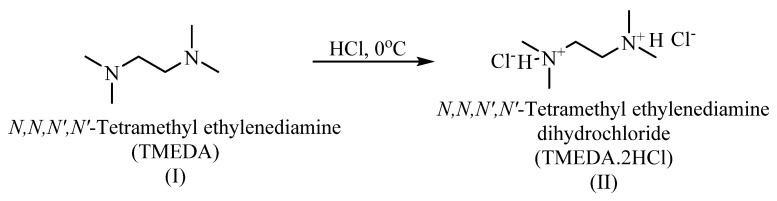
Synthesis of bis-quaternary onium salt-based on *N,N,N′,N′*-tetramethyldiamine (TMEDA).

To a cool *N,N,N′,N’*-tetramethylethylenediamine (6.4 mL) in an ice-bath, concentrated hydrochloric acid (9 mL) was added. The mixture was stirred at 0 °C for one hour, and the stirring was continuing for 24 h at 25 °C. The reaction mixture was diluted with water and then was evaporated using a Rotavapor till dryness to remove excess hydrochloric acid. The product (II) was purified by washing with ether and vacuum dried at 40 °C for 24 h to give 5.2 g (23.80%) (Scheme 1).

### 2.3. Synthesis of Antimicrobial Polymers

#### 2.3.1. PSI: TMEDA.2HCl (1:1) (IV)

**Scheme 2:**
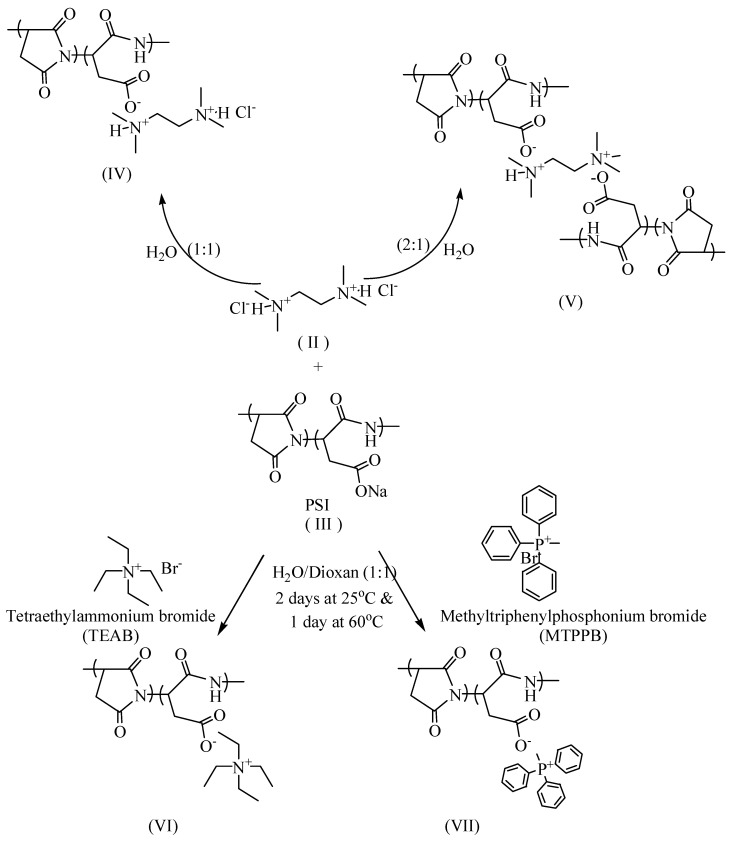
Synthesis of quaternary salts; ammonium and phosphonium compounds.

To a solution of bis-quaternary salt TMEDA.2HCl (II) (10 mmol) in water (20 mL), a solution of the copolymer (III) (10 mmol) in water (20 mL) was added. The reaction mixture was stirred for 48 h at 25 °C and was then heated at 60 °C for extra 24 h. The solvent was concentrated under vacuum, and the product was obtained through precipitation after dropwise addition to excess ethanol. The product (IV) was filtered using sintered glass (G3) and dried at 40 °C in a vacuum oven to give 3.65 g (89.68% yield) (Scheme 2).

The ^1^H NMR (700 MHz, *D*_2_*O*), of compound (IV) showed a signal at δ 2.9 ppm that was assigned to the protons of terminal methyl groups, while the signals at δ 3.5 ppm were attributed to methylene protons of the diamine. The signal at δ 4.71 ppm, most probably due to the overlap of methine protons and residual *D*_2_*O* (Supplementary Material, Figure S1). The ^13^C NMR (700 MHz, *D*_2_*O*), of compound (VI) showed signal at δ 16.7 ppm for the carbons of the tetramethyl group [(CH_3_)_2_ N^+^]. Signals that appeared at δ 36.0 ppm were assigned to the methylene carbons of the succinimide rings. Signals that appeared at δ 43.3–43.4 ppm were assigned to the methylene carbons [α-C of carbonyl C=O and β-C of carbonyl (C=O)-N]. The methine carbons appeared at δ 51–52 ppm. Signals that appeared at δ 57.4 ppm were assigned to the methylene carbons (CH_2_-CH_2_-N^+^). Amide carbonyl carbon appeared at δ 173–174 ppm, and carboxylate carbons appeared at δ 176–177 ppm (Figure S2).

#### 2.3.2. PSI: TMEDA.2HCl (1:0.5) (V)

To a solution of the copolymer (10 mmol) in water (20 mL), a solution of bis-quaternary salt of TMEDA.2HCl (II) (5 mmol) in water (20 mL) was added dropwise within one hour at 25 °C. The reaction mixture was stirred at 25 °C for 48 h and was heated at 60 °C for extra 24 h. The solvent was concentrated under vacuum, and the product was obtained through precipitation after dropwise addition to excess ethanol. The product (V) was filtered using sintered glass (G3) and was dried in a vacuum oven at 40 °C to give 2.5 g (79.67% yield) (Scheme 2).

The ^1^H NMR and ^13^C NMR of (V) were much similar to that of compound (IV). The ^1^H NMR (700 MHz, *D*_2_*O*), showed a signal at δ 2.8 ppm that was assigned to the protons of terminal methyl groups, while the signal at δ 3.5 ppm was attributed for methylene protons of the diamine. The signal at δ 4.71 ppm was most probably due to the overlap of methine protons and residual *D*_2_*O* (Figure S3). The ^13^C NMR (700 MHz, *D*_2_*O*), of compound (VI) showed signals at δ 16.7 ppm that were assigned for the tetramethyl group [(CH_3_)_2_ N^+^]. Signals that appeared at δ 36.0 ppm were assigned to the methylene carbons of the succinimide rings. Signals that appeared at δ 43.3–43.4 ppm were assigned to the methylene carbons [α-C of carbonyl C=O and β-C of carbonyl (C=O)-N]. The methine carbons appeared at δ 51–52 ppm. Signals that appeared at δ 57.4 ppm were assigned to the methylene carbons (CH_2_-CH_2_-N^+^). The amide carbonyl carbon appeared at δ 173–174 ppm, and the carboxylate carbons appeared at δ 176–177 ppm (Figure S4).

#### 2.3.3. Synthesis of Quaternary Onium Salts (VI and VII)

To a solution of quaternary salt (15 mmol) in water/dioxane (1:1) (30 mL), a solution of copolymer (I) (15 mmol) in water/dioxane (20 mL) was added. The reaction mixture was stirred at room temperature for 48 h and was heated at 60 °C for extra 24 h. The solvent was concentrated under vacuum, and the product was obtained through precipitation after dropwise addition to excess ethanol. The product was filtered using sintered glass (G3) and was dried in a vacuum oven at 40 °C to give 2.95 g (62% yield) of the product (VI) and 2.95 g (42% yield) of the product (VII) (Scheme 2).

The ^1^H NMR (700 MHz, *D*_2_*O*), for compound (VI) sample showed a signal at δ 1.1 ppm, which was assigned to terminal methyl groups of the tetrabutylammonium. The signal that appeared at δ 2.61–2.65 ppm was assigned to methylene protons of succinimide rings. The signal that appeared at δ 2.73–2.81 ppm was assigned to methylene protons of CH_2_ alpha to C=O (α-C=O, and β C (C=O)-N]. The signal at δ 4.71 ppm was likely due to the overlap of methine protons and residual *D*_2_*O* (Figure S5). The ^13^C NMR (700 MHz, *D*_2_*O*), of compound (VI) showed signals at δ 6.6 ppm and 57–58 ppm that were assigned for the methyl carbons and the methylene carbons of the tetrabutyl group, respectively. The signal at δ 39.6 ppm was assigned to CH_2_ alpha to C=O. The methine carbons appeared at δ 66.5 ppm. The amide carbonyl appeared at δ 173–174 ppm, and the carboxylate carbons appeared at δ 176–177 ppm (Figure S6).

The ^1^H NMR (700 MHz, *D*_2_*O*), for compound (VII) showed multiplets between δ 2.6 ppm and 3.1 ppm that were assigned to methylene protons of succinimide rings, and the CH_2_ alpha to C=O (α-C=O, and β C (C=O)-N]. The signals at δ 3.7 ppm were assigned to the CH_2_ alpha to C=O. The signal at δ 4.71 ppm most probably due to the overlap of methine protons and residual *D*_2_*O*. The multiplets at δ 7.6–7.7 ppm were assigned for the phenyl ring protons. The amide NH proton of the aspartate could be overlapped with phenyl ring protons (Figure S7). The ^13^C NMR (700 MHz, *D*_2_*O*), of compound (VII) showed terminal methylene carbons at δ 7.81 ppm, while the methylene carbons appeared at δ 16.7 ppm CH_2_ alpha to C=O (α-C=O, and β C (C=O)-N]. The signal at δ 39.7 ppm was assigned for methine carbon (α-C=O, and β C (C=O)-N]. The methylene carbon alpha to C=O appeared at δ 51 ppm. The methine carbon (α-C=O, and β C (C=O)-NH) appeared at δ 59.2 ppm. The phenyl carbons appeared at δ 129–133 ppm (Figure S8) [21,22]. The amide carbonyl appeared at δ 171–173 ppm, and the carboxylate carbons appeared at δ 176–177 ppm.

### 2.4. Characterizations

Determination of elemental microanalyses was done by using a Heraeus instrument, Heraeus Holding GmbH, Hanau, Germany. ^1^H NMR spectra were recorded on a Bruker AV-700 MHz, Bruker BioSpin GmbH, Rheinstetten, Germany. Thermal properties were studied under a nitrogen atmosphere using thermogravimetric analysis (TGA, TA-Q500 System of TA, Pittsburgh, PA, USA; samples of 5–10 mg, at 30–800 °C and at a scanning rate of 10 °C·min^−1^). Fourier-transform infrared spectroscopy (FTIR) was done using a TENSOR 27, Bruker, Billerica, MA, USA.

### 2.5. Antimicrobial Assessment

#### 2.5.1. Tested Microorganisms

In this study, the microorganisms used included both Gram-negative bacteria (*Escherichia coli*, *Pseudomonas aeruginosa*, and *Salmonella typhi*) and Gram-positive bacteria (*Staphylococcus aureus*) in addition to and the fungi (*Candida albicans*, *Aspergillus niger*, *Aspergillus flavus* and *Cryptococcus neoformans*). Bacteria were preserved over nutrient-agar, whereas the fungi were maintained on Sabouraud agar slopes.

#### 2.5.2. Cut Plug (Agar Diffusion) Procedure for Antimicrobial Activity Test Screening

The antimicrobial activity of biocidal polymers was studied by the cut plug method as prescribed by Valgas et al. [23]. The agar plates were prepared by mixing 0.5 mL of the spore’s suspension or freshly prepared cells of microorganisms with 9.5 mL of sterile Sabouraud’s dextrose medium for fungi and 9.5 mL of nutrient-agar medium for bacteria by pouring into the pre-sterililized Petri plates. The plates were kept at 25 °C to solidify the agar. Uniform wells on the agar were made by using 0.7 mm-diameter pre-sterililized cork borer. The known weight of biocidal polymer samples (20 mg) were loaded into the well and were incubated at 27 °C for fungi and at 32 °C for bacteria. After incubation for 24 h, the zones of inhibition were recorded in (mm).

#### 2.5.3. MIC Evaluation of Effective Antimicrobial Biocidal Polymers

Half-fold serial dilutions of selected biocidal polymers were taken into account for the preparation of various concentrations of 6.25, 12.5, 25, 50 and 100 mg/mL, whereas negative control was considered as zero concentration. A previously prepared overnight pure cell suspension of 0.5 mL selected microorganism was mixed with 9.5 mL of agar solution in presterilized test tubes, which were incubated at 27 °C for 72 h for fungi and at 32 °C for 24 h for bacteria. The growth of the microorganism was observed by the optical density values, which were spectrophotometrically measured by (Optima SP-300, Optima Inc., Tokyo, Japan) at 620 nm. The MIC was calculated from the observed results for all selected microorganisms [24].

## 3. Results and Discussion

Polymeric materials having onium salts are highly effective as antimicrobial agents [25,26]. The antimicrobial polymers were acquired through the reaction of PSI copolymer (III) with different ratios of TMEDA.2HCl (II) through ion exchange of the sodium ion from the PSI with quaternary nitrogen ion from TMEDA.2HCl (II), to acquire the antimicrobial polymers (IV and V) as shown in (Scheme 2). The product (IV) was obtained by the reaction of equimolar amount from PSI (III) and TMEDA.2HCl (II). The product (IV) has a free pendent chloride ion. In contrast, the product (V) was obtained from the reaction of two moles of PSI (III) with one mole of TMEDA.2HCl (II). Product (V) is a type of ladder-like structure of bis-quaternary salt in between two linear chains of poly(aspartate-*co*-succinimide). For a comparison study, the copolymer (III) was reacted with simple onium salt of ammonium and phosphonium salts, as shown in (Scheme 2). The structure of the different onium salts (bis-quaternary and simple salts; ammonium and phosphonium) were confirmed by various techniques such as FTIR, NMR, CHN and TGA.

### 3.1. Elemental Analysis Halide Ion Estimation

The copolymer of PSI (III) was quaternized with TMEDA. 2HCl (II) to produce the cationic salts, which were confirmed by elemental analysis and the amount of halide ion was estimated by Voldhard’s method [27]. The results were in good agreement with the calculated values as listed in Table 1.

### 3.2. FTIR Analysis

**Figure 1:**
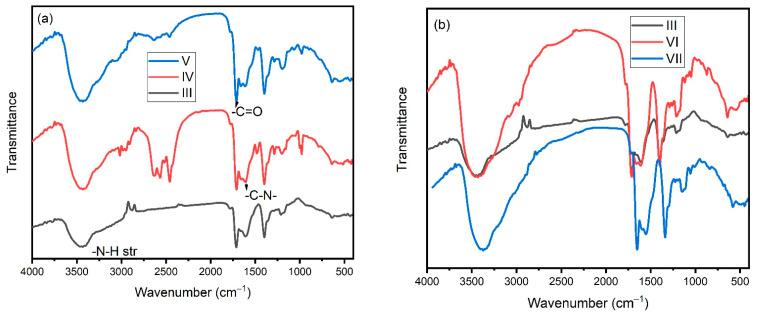
FTIR spectra of polymers (**a**) III, IV and V; and (**b**) III, VI and VII.

FTIR spectra were used to analyze the structure of PSI and bis-quaternary salts (Figure 1). The sharp peak observed at 1715 cm^−1^ in PSI (III) was attributed to the carbonyl group (-C=O). The broad peak at 3450 cm^−1^ was observed for the –N-H stretching of the PSI unit. In the case of binary salt (IV), the absorption peak for the carbonyl group was shifted to a lower frequency like 1708 cm^−1^ and the –N-H stretching was shifted to a lower frequency around 3428 cm^−1^. In comparison between the binary and quaternary ammonium salts of the copolymer, there was no change in peaks except the increase in the sharpness of some peaks was observed. The –CH_2_ symmetric stretching was observed at 2946 cm^−1^ for the PSI unit [28]. The symmetric stretching of –C-N was observed at 1395 cm^−1^. The absorption band at 1608 cm^−1^ and 1715 cm^−1^ were attributed to –C=O of amide and carboxyl carbonyl groups, respectively [28]. The absorption band appeared at 1400 cm^−1^ for symmetric stretching of the –C-N group, which was shifted to higher values. The characteristic bands at 980 cm^−1^, 1195 cm^−1^, and 1282 cm^−1^ corresponded to –C-H bending, -C–C-N bending that was observed in quaternary salts [29].

FTIR spectra showed the absorption band appeared at 1648 cm^−1^ that was corresponding to P-C=C in the triphenylphosphine group [30] present in the phosphonium salts (VII), whereas the same was not observed in ammonium salts (VI). The –N-H stretching was observed for phosphonium salts at 3365 cm^−1^ and 3430 cm^−1^ for ammonium salts. Some of the absorption bands were attributed to ammonium and phosphonium groups due to the N-H and P-H stretching, which may be overlapped with other stretching bands of –OH and –CH groups of PSI unit at 1700 cm^−1^ to 3000 cm^−1^.

### 3.3. Thermal Studies

**Figure 2:**
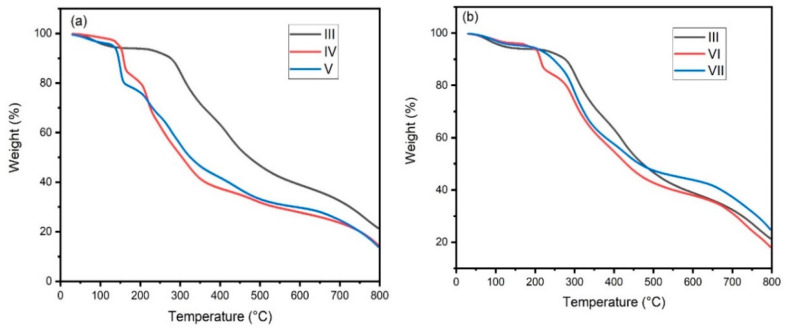
TGA thermogram of polymers (**a**) III–V and (**b**) III, VI and VII.

Table 2 showed the thermal properties of the pure copolymer and its quaternized form, which were studied by thermogravimetric analysis (TGA). All samples undergo a multistep degradation process in which the first step was due to the evaporation of moisture from the temperature range of 30–140 °C (Figure 2a). The degradation of pure PSI started at 283 °C, but in the case of the sample (II) and quaternized salts, the degradation started at 140–206 °C due to the degradation of the additional functional groups attached to the copolymer. The change in the thermal stability was observed by measuring *T*_50_ for pure copolymer around 473 °C, but the quaternized materials loosed 50% of its weight at 305 °C and 326 °C, respectively.

Figure 2b showed variation degradation steps of ammonium and phosphonium-based quaternized salts of the copolymer. The first degradation peak at 206 °C in quaternized ammonium salts was due to the degradation of TEAB, followed by the degradation of PSI at 295 °C. In comparison, the first degradation for the quaternized phosphonium sample was observed at 254 °C, followed by the PSI degradation at 278 °C.

### 3.4. Antimicrobial Assessments

The antimicrobial properties of the various synthesized materials were studied against tested pathogenic bacteria and fungi. The results were compared among the different synthesized compounds used for the quaternization of PSI. The copolymer (III) and its all quaternary forms (IV–VII) were evaluated for its antimicrobial properties against three Gram-negative species, one Gram-positive species and four fungi pathogens. Table 3 showed the inhibition zone values of quaternized compounds in comparison with the control as copolymer (III). The diameters of the inhibition zones in (mm) created by 20 mg of tested polymers (III–VII) against different types of bacteria after 24 h were preserved on nutrient agar slopes and for fungi were maintained on Sabouraud agar slopes for 72 h. The effect of antimicrobial properties was compared with the copolymer (III) as control with all quaternized samples (IV–VII). Antibacterial properties of quaternized salts by TMEDA.2HCl, both ratios IV and V, showed high inhibition zone value, especially for Gram-negative bacteria such as *E. coli, P. aeruginosa* and *S. typhi* when compared with the control. Biocidal polymer (VI) showed an inhibition zone of 14 ± 1.3 (mm) in the case of *S. typhi*, which is the most sensitive bacteria to the tested biocidal (VI). However, *S. aureus* is very sensitive to biocidal (IV), which performed 18 ± 1.7 (mm) against the most affected tested bacteria. On the other hand, biocidal polymer (VII) gave 19 ± 1.5, 18 ± 1.6 and 14 ± 1.3 (mm) against *E. coli, S. aureus* and *P. aeruginosa*, respectively. The comparison between quaternized ammonium and phosphonium salts for antimicrobial effect showed that the phosphonium containing compounds marked activity against fungi, Gram-positive (*S. aureus*) and Gram-negative species (*E. coli*, *P. aeruginosa* and *S. typhi*). The significant difference between the bacterial cell wall structures of both Gram-positive and Gram-negative has an impact on the antibacterial results. In the case of Gram-negative species, the antibiotic must negotiate an outer membrane containing a distinctive lipopolysaccharide–lipoprotein complex that is responsible for inhibiting several antibiotics from reaching cells an otherwise responsive intercellular target. The movement of hydrophilic molecules over Gram-negative bacteria’s lipophilic outer membrane, which includes aqueous transmembrane channels, depends on their ionic charge as well as their molecular size [31]. These channels are used by many antimicrobial agents to have entree to Gram-negative organisms, while other paths are also used [32]. Gram-positive cells, unlike Gram-negative cells, have a peptidoglycan layer that is made up of networks with multiple pores, allowing external molecules to reach the cell with no difficulty [33].

The target site of such cationic biocides has been reported to be the cytoplasmic membranes of bacteria. In the following six stages, the antibacterial process of these cationic disinfectants can be summarized [34]: (a) adsorption to the surface of the bacterial cell; (b) diffusion over a cell wall; (c) binding with cytoplasmic membrane; (d) destruction of the cytoplasmic membrane; (e) potassium ion and other cytoplasmic constituents are released, and finally (f) the precipitation of the cell contents and death of the cell.

Clarification of the individual roles of bacteria membrane, permeabilization and depolarization, is important to understand the mode of action of the antimicrobial agents as well as providing evidence for the oddity of membrane integrity in bacterial viability. Moreover, for Gram-negative bacteria, understanding the role of the outer membrane in preventing the access of antimicrobial agents to the cytoplasmic membrane is important. In order to estimate the efficiency of the prepared polymers, the cytoplasmic membrane of Gram-negative bacteria was depolarized, inducing solute leakage, and causing a bactericidal effect remains to be tested.

Based on the observed activity, two fungi and two bacteria were selected and further evaluated by the method of minimum inhibitory concentration (MIC). The MIC values of the selected fungi and bacterial pathogens are listed in (Table 4). The MIC was used in two different concentrations, which are 25 mg/mL and 50 mg/mL.

### 3.5. Effect of TMEDA Quaternized Copolymer

**Figure 3:**
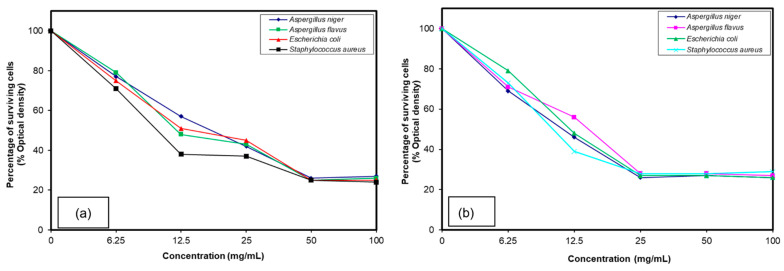
Growth inhibition against the various microorganisms examined of different polymer concentrations (**a**) IV and (**b**) V.

MIC values showed that 6.25 mg/mL of compound (IV) kills around 23% of *A. niger*, which was increased to 73% in 50 mg/mL; upon increasing the concentration, the killing ratio of fungi was not affected, and a similar trend was observed in other species of fungi and bacteria (Figure 3). However, in the case of compound (V), MIC values showed that 6.25 mg/mL kills 31% of *A. niger* and 25 mg/mL kills 74%. The difference between these two salts is the linkage of PSI copolymer on either one side of the nitrogen atom from the TMEDA or both sides. Among the results of MIC values, compound (V) was found more effective than compound (IV) while killing the pathogenic microbial strains, which was due to the copolymer linkage on both sides of TMEDA.

### 3.6. Comparison between the Ammonium and Phosphonium Salts

**Figure 4:**
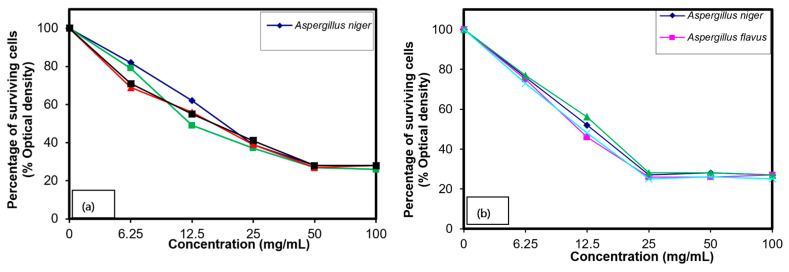
Growth inhibition against the various microorganisms examined of different polymer concentrations (**a**) VI and (**b**) VII.

Figure 4 showed the percentage of surviving cells against the different concentrations of ammonium and phosphonium-based quaternized copolymers. Percentage of cell surviving showed that 6.25 mg/mL concentration of the compound (VI) kills 18% of *A. niger*, 21% of *A. flavus*, 29% of *E. coli* and 30% of *S. aureus*. Upon increasing the concentration, the killing ratio increased gradually to 50 mg/mL and further addition more than 50 mg/mL does not have any effect. However, in the case of phosphonium quaternized copolymer (VII), 6.25 mg/mL concentration kills 24% of *A. niger*, 25% of *A. flavus*, 23% of *E. coli* and 27% of *S. aureus*. The killing ratio was increased while increasing the concentration to 25 mg/mL; further addition of more than this level did not kill a greater percent of pathogens.

Phosphonium-based quaternary copolymers showed better results of killing pathogens with a low concentration of 25 mg/mL, which is in good agreement with a zone of inhibition observed. These results are comparable to that obtained by Kenawy et al. [35] while comparing the antimicrobial effect of ammonium and phosphonium-based modified copolymer.

## 4. Conclusions

Water-soluble copolymer-based on poly(aspartate-*co*-succinimide) containing quaternary salts were prepared by using different sources like TMEDA, TEAB and MTPPB. The successful formation of onium salts was confirmed by FTIR spectra results via shifting of carbonyl group’s position to lower values, and the presence of new absorption bands, e.g., -C-Cl, –N-H stretching for ammonium salts and P-C=C, P-H stretching in triphenylphosphine group present in the phosphonium salts. The NMR spectra of onium compounds revealed the presence of terminal methyl group of tetrabutylammonium (VI) and methylene proton of succinimide ring (VII). The formation of onium salts was further confirmed by elemental microanalysis, which showed that the observed values are close to the calculated values. The formed onium salts (IV-VII) showed low thermal stability compared to the PSI copolymer due to the degradation of the additional functional groups attached to the copolymer. Comparing phosphonium compounds with those of ammonium compounds showed a marked activity against Gram-negative species (*E. coli*, *P. aeruginosa* and *S. typhi*), Gram-positive species (*S. aureus*) and fungi. The results of the antimicrobial activity were compared with the copolymer alone and its quaternary salts, which showed high inhibitory activity against the tested microorganisms in all pathogenic species. The MIC of the selected species was evaluated, and the results showed that 25 mg/mL was the optimum concentration for (VII and V) and 50 mg/mL for (VI and IV). The interpretation of the obtained antimicrobial results indicated that the phosphonium salts are more effective than ammonium salts.

## Figures and Tables

**Table 1:** Elemental analysis of copolymer and its quaternized salts.

Sample Code	Carbon (%)		Hydrogen (%)		Nitrogen (%)		Halide (%)	
	Calc.	Found	Calc.	Found	Calc.	Found	Calc.	Found
III	44.05	36.76	3.23	2.92	12.84	9.92	-	-
IV	42.85	38.85	6.25	7.20	16.66	10.93	9.72	7.33
V	46.87	39.44	5.46	5.22	16.40	10.91	-	0.33
VI	56.14	37.53	7.89	4.17	12.28	8.69	-	-
VII	66.25	37.44	5.11	3.77	5.72	8.84	-	-

**Table 2:** Thermogravimetric parameters polymers (III–VII).

Polymer Code	*T*_on_ (°C)	*T*_50_ (°C)	Residue (at 600 °C)
III	283	473	39
VI	206	431	38
VII	254	465	44
IV	150	305	27.9
V	140	326	29.7

**Table 3:** Inhibition zone diameters (mm) for quaternized salts of copolymers.

Tested Microorganism	Diameter (mm) of the Inhibition Zone *				
	Control	Biocidal Polymers			
	III	VI	VII	IV	V
*Candida albicans*	10 ± 1.0	12 ± 1.2	14 ± 1.3	13 ± 1.2	13 ± 1.1
*Cryptococcus neoformans*	9± 0.8	13 ± 1.1	13 ± 1.2	14 ± 1.3	12 ± 1.2
*Aspergillus niger*	9 ± 0.7	16 ± 1.4	21 ± 2.0	17 ± 1.5	21 ± 2.0
*Aspergillus flavus*	11 ± 1.0	18 ± 1.7	20 ± 1.9	19 ± 1.8	20 ± 1.8
*Escherichia coli*	10 ± 0.8	16 ± 1.5	19 ± 1.5	16 ± 1.5	21 ± 1.7
*Staphylococcus aureus*	8 ± 0.8	17 ± 1.6	18 ± 1.6	18 ± 1.7	17 ± 1.5
*Pseudomonas aeruginosa*	9 ± 0.6	12 ± 1.2	14 ± 1.3	13 ± 1.2	11 ± 1.0
*Salmonella typhi*	8 ± 0.7	14 ± 1.3	13 ± 1.2	11 ± 1.0	12 ± 1.0

* Average ± standard deviation.

**Table 4:** Minimal inhibitory concentrations (MIC; mg/mL) of selected polymers against different susceptible microorganisms.

Tested Microorganism	MIC (mg/mL) of Biocidal Polymers			
	VI	VII	IV	V
*Aspergillus niger*	50	25	50	25
*Aspergillus flavus*				
*Escherichia coli*				
*Staphylococcus aureus*				

## Data Availability

The data presented in this study are available on request from the corresponding author.

## Supplementary Materials

The following are available online at https://www.mdpi.com/2073-4360/13/1/23/s1, Figure S1: ^1^HNMR of copolymer (IV), Figure S2: ^13^CNMR of copolymer (IV), Figure S3: ^1^HNMR of copolymer (V), Figure S4: ^13^CNMR of copolymer (V), Figure S5: ^1^HNMR of copolymer (VI), Figure S6: ^13^CNMR of copolymer (VI), Figure S7: ^1^HNMR of copolymer (VII), Figure S8: ^13^CNMR of copolymer (VII).
