# Supplementary material for: Biocidal Polymers: Synthesis, Characterization and Antimicrobial Activity of Bis-Quaternary Onium Salts of Poly(aspartate-co-succinimide)
Source: Polymers (Basel). 2020 Dec 23;13(1):23. doi: 10.3390/polym13010023 (PMC7793505; doi:10.3390/polym13010023)
Supplement: Supplementary file 1 [file polymers-13-00023-s001.pdf]

Article

Biocidal Polymers: Synthesis, Characterization and Antimicrobial Activity of Bis-Quaternary Onium Salts of Poly(aspartate-*co*-succinimide)

Mohamed H. El-Newehy[1,2]*, Meera Moydeen A[1], Ali K. Aldalbahi[1], Badr M. Thamer[1], Yehia A.-G. Mahmoud[3] and Hany El-Hamshary[1,2]

[1] Department of Chemistry, College of Science, King Saud University, Riyadh 11451, Saudi Arabia.
[2] Department of Chemistry, Faculty of Science, Tanta University, Tanta 31527, Egypt.
[3] Department of Botany and Microbiology, Faculty of Science, Tanta University, Tanta 31527, Egypt.
* Corresponding author: E-mail: melnewehy@ksu.edu.sa; Tel.: +966-11-4675894
Received: 29 October 2020; Accepted: date; Published: date

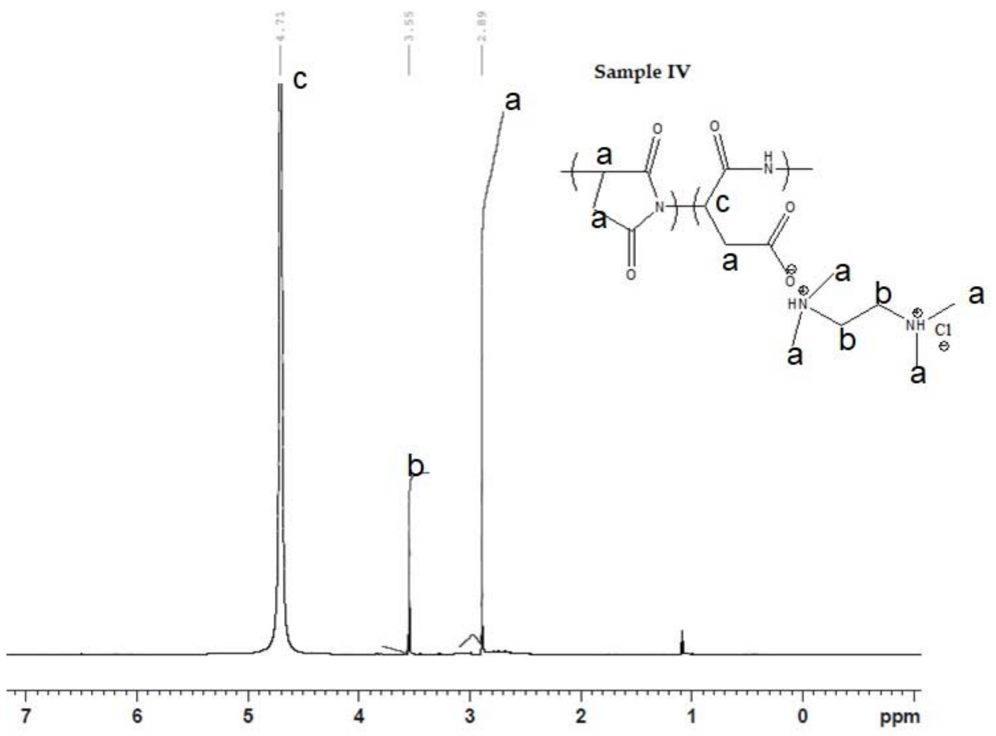

Figure S1. ^{1}HNMR of copolymer (IV).

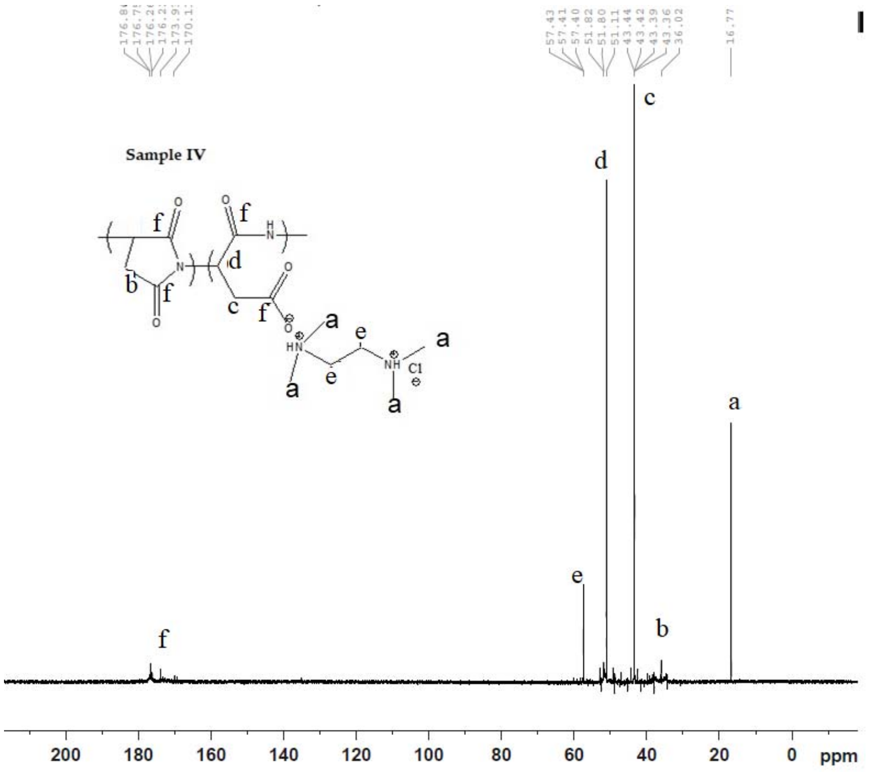

Figure S2. ^{13}CNMR of copolymer (IV).

Sample V

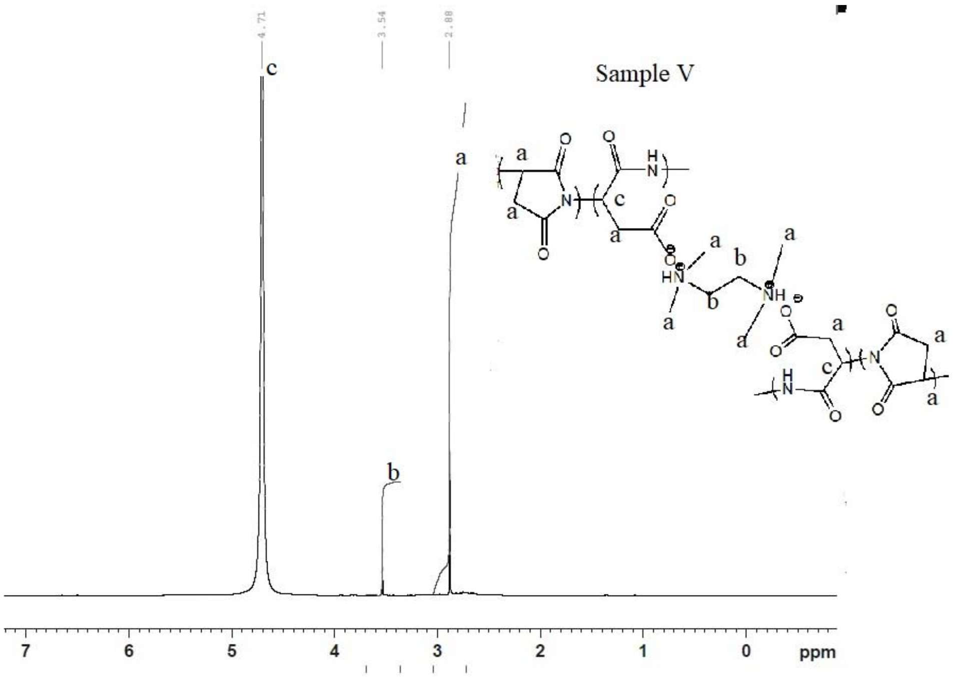

Figure S3. ^{1}HNMR of copolymer (V).

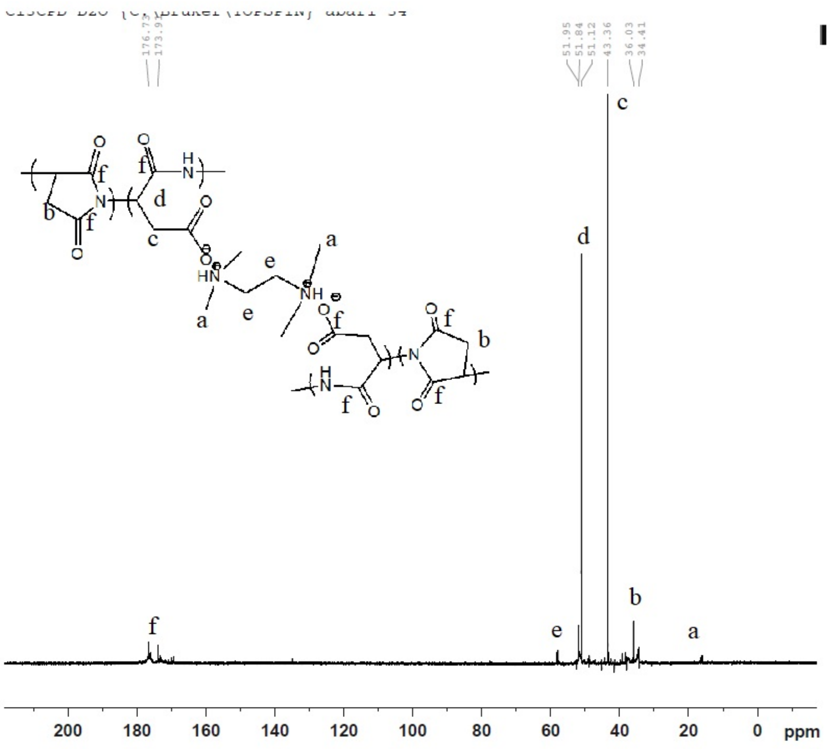

Figure S4. ^{13}CNMR of copolymer (V).

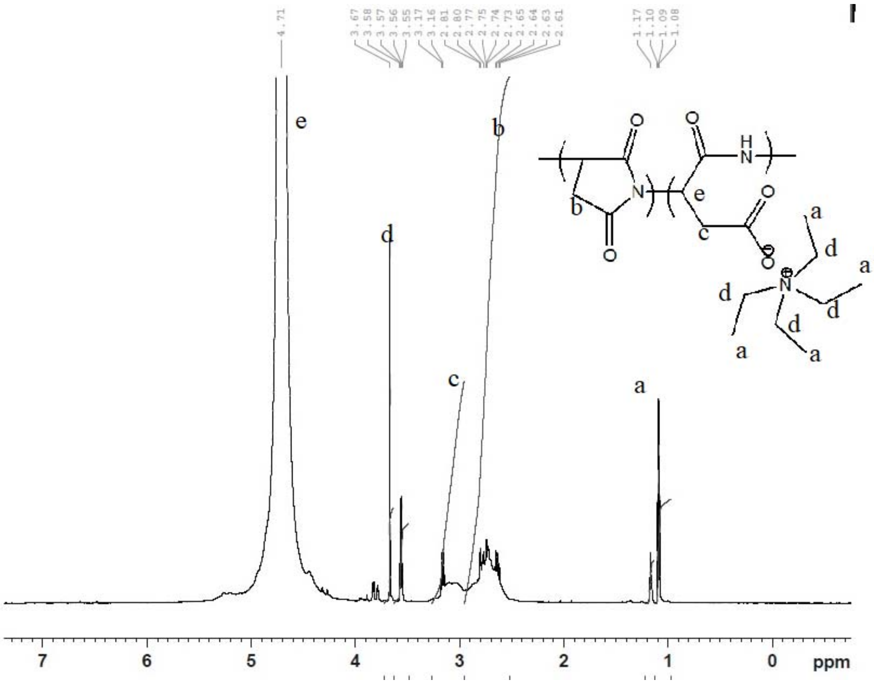

Figure S5. ^{1}HNMR of copolymer (VI).

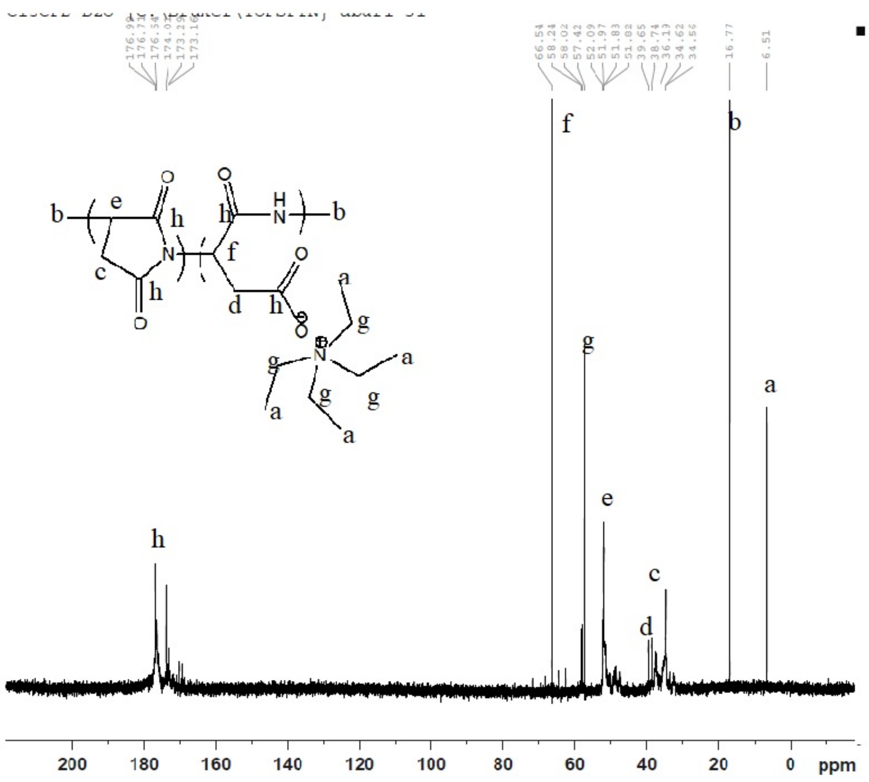

Figure S6. ^{13}CNMR of copolymer (VI).

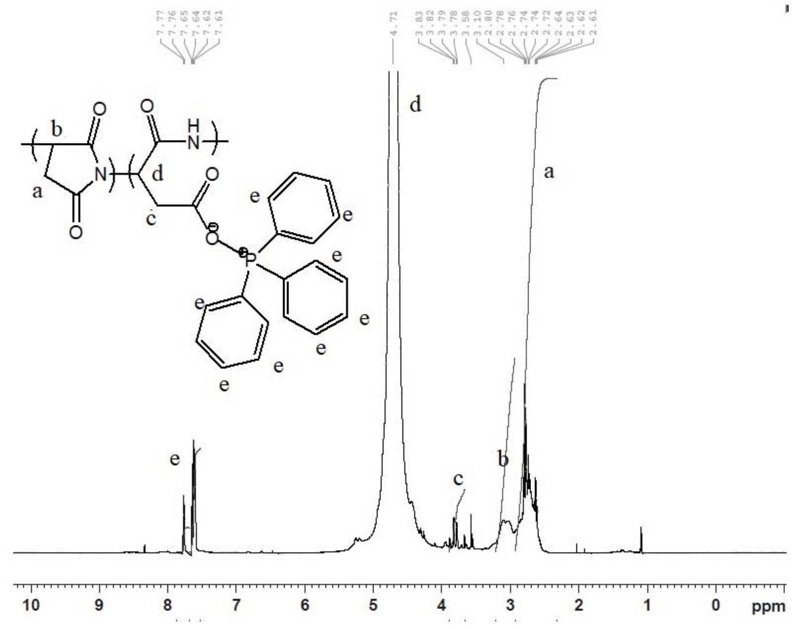

Figure S7. ^{1}HNMR of copolymer (VII).

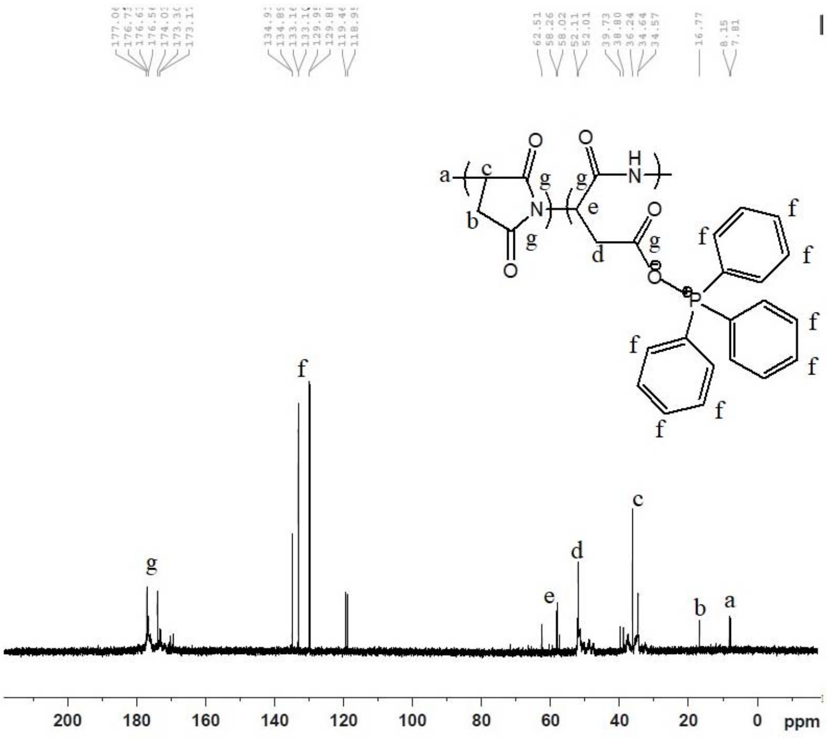

Figure S8. ^{13}CNMR of copolymer (VII).

Publisher's Note: **MDPI stays neutral with regard to jurisdictional claims in published maps and institutional affiliations.**

© 2020 by the authors. Submitted for possible open access publication under the terms and conditions of the Creative Commons Attribution (CC BY) license (http://creativecommons.org/licenses/by/4.0/).
